# Diet Quality and Dietary Intake in Breast Cancer Survivors Suffering from Chronic Pain: An Explorative Case-Control Study

**DOI:** 10.3390/nu16223844

**Published:** 2024-11-09

**Authors:** Sevilay Tümkaya Yılmaz, Ömer Elma, Jo Nijs, Peter Clarys, Iris Coppieters, Tom Deliens, Patrick Calders, Eline Naert, Anneleen Malfliet

**Affiliations:** 1 Pain in Motion Research Group (PAIN), Department of Physiotherapy, Human Physiology and Anatomy, Faculty of Physical Education and Physiotherapy, Vrije Universiteit Brussel, 1090 Brussels, Belgium; sevilay.tumkaya.yilmaz@vub.be (S.T.Y.);; 2 Pain in Motion International Research Group, 1090 Brussels, Belgium; oelma@bournemouth.ac.uk; 3 Physiotherapy Unit, Department of Rehabilitation and Sport Sciences, Faculty of Health and Social Sciences, Bournemouth University, Bournemouth BH8 8GP, UK; 4 Department of Physical Medicine and Physiotherapy, University Hospital Brussels, 1090 Brussels, Belgium; 5 Unit of Physiotherapy, Department of Health and Rehabilitation, Institute of Neuroscience and Physiology, Sahlgrenska Academy, University of Gothenburg, 405 30 Gothenburg, Sweden; 6 Department of Movement and Sport Sciences, Faculty of Physical Education and Physiotherapy, Vrije Universiteit Brussel, 1050 Brussels, Belgium; 7 Experimental Health Psychology Research Group, Faculty of Psychology and Neuroscience, Maastricht University, 6211 LK Maastricht, The Netherlands; 8 The Laboratory for Brain-Gut Axis Studies (LaBGAS), Translational Research Center for Gastrointestinal Disorders (TARGID), Katholieke Universiteit, 3000 Leuven, Belgium; 9 Department of Rehabilitation Sciences, UGhent-Ghent University, 9000 Ghent, Belgium; 10 Department of Medical Oncology, Ghent University Hospital, 9000 Ghent, Belgium; 11 Research Foundation Flanders (FWO), 1000 Brussels, Belgium

**Keywords:** breast cancer survivors, chronic pain, diet, inflammation

## Abstract

**Background/Objectives:** Dietary factors may significantly influence pain management in cancer survivors. However, a substantial gap exists regarding the relationship between nutrition and chronic pain in this population. This study examined differences in diet quality and dietary intake between breast cancer survivors (BCS) experiencing chronic pain and healthy controls (HC). It also aimed to understand the associations between dietary elements and pain-related outcomes within the BCS group. **Methods**: A case-control study was conducted with 12 BCS experiencing chronic pain and 12 HC (ages 18–65). Data collection included body composition, experimental pain assessments, pain-related questionnaires, and a 3-day food diary to calculate diet quality using the Healthy Eating Index-2015 (HEI-2015) and Dietary Inflammatory Index (DII). Statistical analyses evaluated group differences and associations between dietary factors and pain within the BCS group. **Results:** There were no significant differences in HEI-2015 scores between BCS and HC, but BCS had a significantly lower DII score (*p* = 0.041), indicating a more anti-inflammatory diet. BCS also showed higher intake of omega-3, vitamins B6, B12, A, D, and magnesium (*p* < 0.05). While total diet quality scores did not correlate with pain outcomes, several HEI-2015 and DII components, such as dairy, sodium, protein, vitamin C, and vitamin D, showed moderate positive or negative correlations with pain measures. **Conclusions**: Despite no overall differences in diet quality, BCS with chronic pain consumed more anti-inflammatory nutrients than HC. Complex correlations between specific dietary components and pain outcomes emphasise the need for further research to explore these links for chronic pain management in BCS.

## 1. Introduction

While the global incidence rate of breast cancer is rising, the number of breast cancer survivors (BCS) and their survival durations are also increasing due to advancements in early detection programs and treatments [1]. Despite the transition of many breast cancer patients into survivors, a considerable proportion continues to experience late effects, including chronic pain (e.g., aromatase inhibitor-induced musculoskeletal pain, pain at the surgery or radiation site), which is one of the most challenging side effects of treatment [2]. Despite the available evidence supporting the efficacy of various pain therapies, survivors’ needs are frequently unmet [3].

Over the past decade, there has been a stronger focus on dietary and nutritional variables in chronic pain management [4,5,6]. While the association between diet and breast cancer risk [7,8], prognosis and recurrence [9,10], and quality of life of BCS [11] has been recognised, a substantial knowledge gap remains in understanding the relationship between nutrition and chronic pain experienced by BCS [12].

Chronic pain often arises from a persistent pro-inflammatory state [13], which is also associated with the increased risk of various cancers, including breast cancer [14]. Notably, pro-inflammatory diets are linked to increased pain sensitivity in some other persistent pain populations, like fibromyalgia [15] and low back pain [16]. Inflammation in BCS has been associated with diet quality in multiple studies [17,18]. Furthermore, emerging research claims that diet quality may influence quality of life, including bodily pain in BCS [19]. Nevertheless, the direct investigation of the association between diet quality and chronic pain in this population remains unexplored.

Furthermore, several studies propose that individuals who suffer from chronic pain may benefit from the consumption of food and micronutrients that have anti-inflammatory features [13,20]. It is well known that after a cancer diagnosis, the majority of people modify their diet, with some changes proving beneficial (e.g., increased fruit and vegetable intake, reduced meat and alcohol consumption), while others lacked evidence-based support (e.g., avoiding chicken, seafood, and carbohydrates) [21]. Additionally, more than half of survivors report taking dietary supplements [21]. According to a systematic analysis, BCS are more likely to consume supplements (ranging from 67 to 87% for any vitamin or mineral) compared to other cancer survivors [22]. Although strategically choosing anti-inflammatory foods/nutrients while avoiding pro-inflammatory foods/nutrients may potentially improve pain-related issues in BCS, concerns remain regarding the other nutritional factors. Therefore, a detailed investigation of diet quality and specific dietary components (like fruit consumption and micronutrients) could offer valuable insights into the broader understanding of this important and topical issue.

To address these knowledge gaps, this study primarily aimed to examine the differences in diet quality and dietary intake among BCS experiencing chronic pain in comparison to a cohort of pain-free healthy controls (HC). Subsequently, the study aimed to unravel associations between dietary factors and pain-related outcomes (including pain sensitivity, endogenous analgesia, and self-reported measures) within the BCS population with pain.

## 2. Methodology

### 2.1. Study Design

This explorative case-control study was performed as part of the baseline assessment of a randomised cross-over trial (clinicaltrials.gov NCT04459104) and reported following the STROBE checklist for observational studies [23]. Data collection was performed between September 2020 and March 2023 at Vrije Universiteit Brussel, KU Leuven and Ghent University following approval by the Medical Ethics Committee of the University Hospitals (UZ); UZ Brussels (BUN1432020000025), UZ Leuven (S64298) and UZ Ghent (BC-11029).

### 2.2. Participants

This case-control study included both women suffering from chronic pain post-breast cancer as well as healthy, pain-free women. Female, Dutch-speaking BCS aged 18 to 65 years old suffering from chronic pain (at least 3 months and at least 3 days per week [24]) who completed primary cancer treatment at least 3 months prior to study participation [25] were included in the study. Participants had to abstain from using analgesics, nicotine, caffeine, and alcohol 48 h before assessments. The study excluded BCS with additional systemic diseases (such as hypertension or type 2 diabetes), recent injuries, severe psychological or mental disorders, cognitive impairment, pregnancy, new tumours, or metastases.

The study included female, Dutch-speaking HC aged 18 to 65 years old, with no known health condition, no analgesics/nicotine/caffeine/alcohol consumption 48 h prior to the assessments, no current pregnancy, and no history of pregnancy in the previous year.

### 2.3. Setting

Study participants were recruited through the distribution of posters and flyers at UZ Brussel, UZ Leuven, and UZ Ghent, as well as to general medical centres, pharmacies, private physiotherapy clinics around Brussels, and social media channels. Eligible candidates were invited for an assessment at Vrije Universiteit Brussel, UZ Leuven, or Ghent University, depending on their location of preference. Prior to any study-related procedure, each participant was asked to read and sign a participant information and consent form.

**Figure 1:**
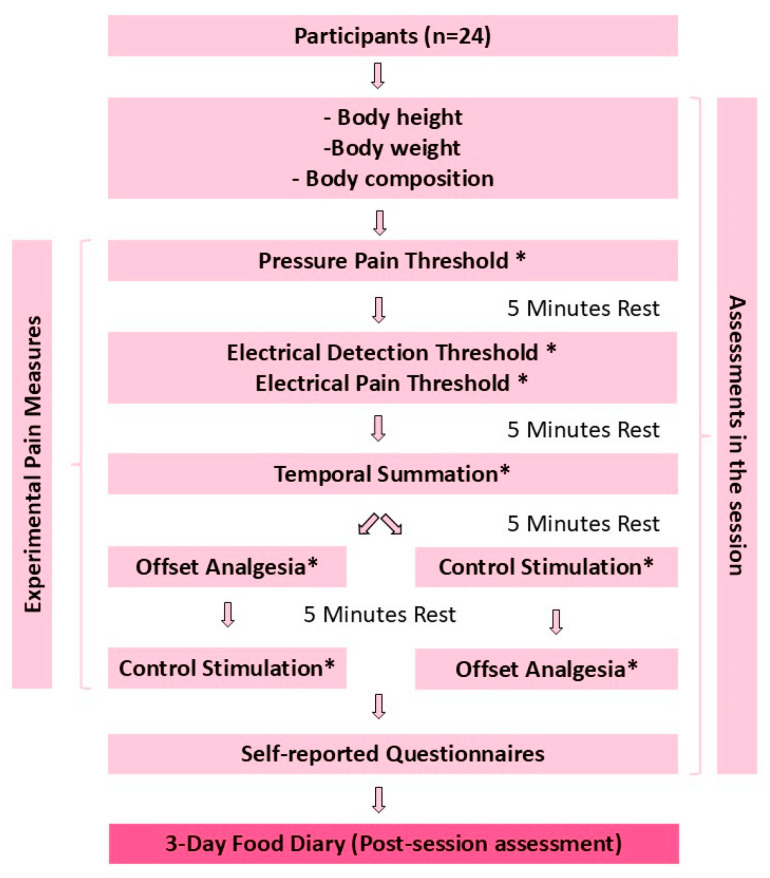
Flow of the study. * Test side changes were separated by a 30-s rest period within the measurement.

This case-control study includes data from a single assessment session (Figure 1). Two assessors collected the data, with each assessing half of the case and control participants. The assessment included measurements of body height, weight, and body composition, as well as experimental pain assessments. Next, individuals were asked to complete self-reported questionnaires such as the Brief Pain Inventory (BPI), Douleur Neuropathic-4 (DN4) Questionnaire, and Central Sensitization Inventory (CSI). Participants were given a 3-day food diary with clear instructions to complete and return it within two weeks of the assessment session.

### 2.4. Outcome Measurement Tools

#### 2.4.1. Sample Characteristics

Each participant’s age, weight, height, cancer stage, affected site from cancer, history of cancer treatment (surgery, chemotherapy, radiotherapy, endocrine therapy or immunotherapy), years after treatment, dominant pain site, physical activity level (in metabolic equivalent (MET) minutes/week), and quality of life scores were collected. Physical activity level was assessed by the International Physical Activity Questionnaire (IPAQ), a reliable and validated measure [26], used in various BCS studies [27,28,29]. Health-related quality of life was assessed with the validated Short Form-36 Health Survey (SF-36) [30,31], with higher scores indicating better health [32].

#### 2.4.2. Dietary Measures

The dietary intake of participants was assessed by using a 3-day food diary [33]. To account for any potential day-of-the-week effects, two weekdays and one weekend day were included [34]. A “sample page” and comprehensive verbal instructions for keeping track of daily food intake were provided. Completed records were analysed by a validated automated self-administered dietary assessment software database (ASA24, 2023, National Cancer Institute) [35]. Individual dietary intakes and diet quality were calculated using dietary data derived from the analysis of a 3-day food diary [16,36,37].

##### Healthy Eating Index-2015

The Healthy Eating Index (HEI)-2015 is an assessment tool to assess dietary quality [38]. It has 13 items in total [38]. The HEI-2015 identifies nine items as “Adequacy Components”, which are considered healthy: total fruit (all forms, including fruit juice), whole fruit (all forms except fruit juice), vegetables, greens and beans, whole grains, dairy, total protein foods, seafood and plant proteins, and fatty acids [39]. The remaining four items, referred to as “Moderation Components” (refined grains, sodium, added sugars, and saturated fats), should be limited. The HEI-2015 scores can vary from 0 to 100, with higher scores representing higher diet quality.

##### Dietary Inflammatory Index

The Dietary Inflammatory Index (DII) was designed to determine the inflammatory potential of a diet [40]. In total, 45 dietary parameters were reported to have significant effects on systemic inflammation after reviewing around 6500 publications on the six inflammatory biomarkers (interleukin (IL)-1β, IL-4, IL-6, IL-10, tumour necrosis factor-alpha (TNF-α), and C-reactive protein(CRP)) [40]. In this case-control study, the DII score was calculated using 28 of the above 45 parameters—including kcal, protein, fat, carbohydrates, alcohol, caffeine, fibre, cholesterol, saturated fatty acids, monounsaturated fatty acids, polyunsaturated fatty acids, omega-3 polyunsaturated fatty acids, omega-6 polyunsaturated fatty acids, vitamin A, C, D, E, B1, B2, B3, B6, B9, B12, beta-carotene, iron, magnesium, zinc, and selenium—that were computed from the 3-day dietary diary. DII scores calculated from 28 food parameters usually range between −5.5 and +5.5 [41]. A negative DII score indicates an anti-inflammatory diet, whereas a positive score indicates a pro-inflammatory diet.

##### Additional Component—Water

According to the International Association for the Study of Pain’s (IASP) 2019 fact sheet on ‘Nutrition and Chronic Pain’, dehydration may increase pain sensitivity [42]. Since water consumption is not included in any of the two indices, we included water intake (from 3-day dietary diary) as an additional component in the study to provide a more comprehensive dietary investigation.

#### 2.4.3. Pain Measures

In the experimental pain measures, to prevent contamination, all the experimental pain assessments were separated by a 5-min rest period. To eliminate sequence bias, the order of test locations was randomised by having participants draw envelopes containing the names of the test sites one by one. Similarly, the sequence of stimuli for the Offset Analgesia test (offset analgesia; control stimulation) was randomised using the same method.

##### Experimental Pain Measures

Pressure Pain Threshold (PPT)

Pressure pain threshold (PPT) is defined as the minimum amount of pressure at which a sensation of pressure transforms into pain [43]. PPT was assessed using a digital pressure algometer with a 1 cm^2^ tip (Wagner Instruments, Greenwich). The pressure was gradually increased at a rate of one kilogram per second (kg/s). When the participants experienced the pressure as painful, they were instructed to say “stop” [44]. PPT was assessed bilaterally, since bilateral hyperalgesia occurs in BCS, at the musculus pectoralis major, which has been suspected of primary (hyper)algesia in BCS [45,46], as well as the tibialis anterior, a distant reference point. Two measurements were taken from each location, separated by a 30-s interval, and averaged to limit measurement error, using the mean PPT value (kg/cm^2^) for analysis.

Electrical Detection and Electrical Pain Thresholds

Electrical detection threshold (EDT) and electrical pain threshold (EPT) were measured with the Surpass LT stimulator (EMS Biomedical, Korneuburg, Austria) at the sural nerve and median nerve, at both sides [47].

The median nerve test location involved applying a bipolar felt pad electrode to the skin overlying the median nerve, with the cathode 5 cm proximally from the wrist and the anodal 3 cm distally. The sural nerve test location involved placing the cathode 2 cm posterior to the lateral malleolus at the sural nerve innervation area, and the anode was positioned 2 cm distal to the cathode along the nerve’s pathway.

Each stimulus was a constant current rectangular pulse train of five pulses delivered at a frequency of 250 Hz. Stimulation started at 0 mA and gradually increased in 0.5 mA increments until the participant reported the stimulation as faint (=EDT) and then painful (=EPT). The measurements were collected three times, with 30 s intervals in between, and the mean of the three measurements was used in the analysis.

Temporal Summation (TS)

Temporal summation of electrical stimuli was used to determine endogenous pain facilitation, with the same four test sites and randomisation used for EDT and EPT. The study assessed temporal summation by administering 20 electrical stimuli at the calculated EPT intensity and assessing pain levels using a verbal numeric rating scale (VNRS), with scores ranging from 0 (=no pain) to 100 (=worst possible pain) at the first, tenth and twentieth stimuli. The differences between the 10th and 1st VNRS scores, the 20th and 10th VNRS scores, and the 20th and 1st VNRS scores were used as outcome measures for temporal summation.

Electrical Offset Analgesia (OA)

Offset analgesia is an inhibitory paradigm characterised by a disproportionately large decrease in pain sensation following a minor noxious stimulus offset [48]. To measure offset analgesia, a constant current stimulator has been used to apply electrical stimuli as a series of rectangular pulses (frequency: 100 Hz; pulse duration: 1 ms) [49]. The test location was 3 cm distal to the elbow joint on the volar side of the forearm in both the dominant arm and non-dominant arm, based on the validated protocol of Petersen et al. [49]. First, the EPT at the sites was assessed using this current (which is different from that used in other EPT measurements), following the same procedure. Then, the intensity of the stimulation was calculated using the EPT. Participants received painful stimuli at three intervals and intensities: T1 (5 s at 150% EPT), T2 (5 s at 180% EPT), and T3 (20 s at 150% EPT). To ensure safety, the maximum stimulation current was set to 50 mA. In addition, a 30-s control electrical stimulus of 150% EPT was given. Participants were told to rate their VNRS score from 0 (no pain) to 100 (worst possible pain) every 5 s (at 4, 9, 14, 19, 24, and 29 s) throughout each application [49].

##### Self-Reported Pain Questionnaires

Brief Pain Inventory (BPI)

The Brief Pain Inventory (BPI) is a frequently used self-reported pain questionnaire for cancer patients [50]. For the purpose of this study, pain interference and pain severity scores are used [51]. The National Comprehensive Cancer Network’s 2019 recommendations classified responses regarding pain severity into four categories: absence of pain (score of 0), mild pain (score of 1 to 3), moderate pain (score of 4 to 7), and severe pain (score of 8 to 10) [52,53]. Pain interference is a score between 0 (no interference) and 10 (total interference), which reflects how much pain has disrupted daily activities, including general activity, mood, walking ability, work, relationships, sleep, and enjoyment of life [54]. The BPI has acceptable internal consistency, acceptable to excellent test-retest reliability, satisfactory to good construct and criterion validity, and is change-sensitive [50,51].

Central Sensitisation Inventory (CSI)

The Central Sensitisation Inventory (CSI) is a self-reported questionnaire to assess the severity of central sensitisation-related symptoms (Part A) and to screen for central sensitivity syndromes (Part B) [55]. Part A assesses 25 indications on a scale of 0 (never) to 4 (always), with an overall score of 0 to 100 (higher scores indicate more frequent or severe symptoms). The study did not include Part B, which questions central sensitisation-related disorders or specific conditions. A cut-off value of 40 out of 100 yields good sensitivity (81%) and specificity (75%) [56,57]. The CSI’s construct validity, internal consistency, and high reliability have all been demonstrated [55,58].

Douleur Neuropathique 4 (DN4)

The DN4 questionnaire was created as a clinic-based tool for identifying patients with pain generated predominantly by a neuropathic mechanism [59]. The 10-item questionnaire assesses pain by scoring descriptors and indicators as yes (1) or no (0), with a maximum score of 10 [60]. Seven items related to pain quality (i.e., sensory and pain descriptors) are based on the patient’s self-report, while three items are derived from the clinical sensory examination performed by the assessor [61]. The sensory examination of the dominant pain site included testing light touch with cotton, assessing allodynia with a brush, and evaluating pinprick sensation with a pin. The DN4 questionnaire has shown excellent specificity (90%) and sensitivity (83%) in identifying chronic pain linked to a nervous system injury, with a score ≥ 4 serving as the diagnosis of neuropathic pain [59,62].

#### 2.4.4. Anthropometrics

##### Body Height

The Seca 213 stadiometer (Seca GmbH, Hamburg, Germany) was used for measuring body height.

##### Body Composition

Body weight and body composition were assessed using a bioelectrical impedance analysis device, which is a valid method for estimating body composition [63]. The TANITA (MC780MA, Tanita Corp., Tokyo, Japan) was used in this study for this analysis [64]. This is a professional tool that can measure more than 20 parameters [64]. The assessor entered the participants’ sex, age, and height into the device and then instructed them to step barefoot onto the metallic electrodes on the platform and grasp the handles with both hands. The analysis was based on parameters including body weight, body mass index (BMI), body fat percentage, fat-free mass, and total body water percentage.

### 2.5. Statistical Analyses

IBM SPSS Statistics version 29.0.1.1 (244) (IBM Corp., Armonk, NY, USA) was used for the statistical analyses. Categorical data were described as frequency and percentage, whereas continuous data were described as mean and standard deviation (SD) or median and interquartile range (IQR) [65]. *p*-values ˂ 0.05 were considered statistically significant [66].

The Shapiro–Wilk test, histogram, and Q-Q plot [65] were used to determine the normality of distribution. Differences in sample characteristics between the case and control groups were examined using the Mann–Whitney U test or the independent *t*-test. Additionally, Mann–Whitney U and independent *t*-tests were used to evaluate differences in overall diet quality scores (based on HEI-2015 and DII), HEI-2015 components and DII components between BCS with chronic pain and pain-free HCs. Lastly, Pearson correlation coefficient (for parametric testing) and Spearman’s correlation coefficient (for non-parametric testing) [67] were used to assess the correlations between overall diet quality scores (based on HEI-2015 and DII), HEI-2015 components, DII components, and pain sensitivity outcome measures for BCS with chronic pain. The correlation coefficients (r/r_s_) range from −1.00 to 1.00, with r/r_s_ > 0 indicating a positive link, r/r_s_ < 0 indicating a negative relationship, and r/r_s_ = 0 indicating no relationship [68]. The relationship’s strength can be specified as very weak for values between 0.00 and 0.19, weak for 0.20 to 0.39, moderate for 0.40 to 0.70, strong for 0.70 to 0.90, and very strong for >0.90 [68].

## 3. Results

This study included 12 BCS with chronic pain (mean age 52.8 ± 6.3) years and 12 healthy pain-free controls (mean age 47.5 ± 3.8 years). Table 1 illustrates the characteristics of BCS.

The characteristics of the individuals and the differences between groups are summarised in Table 2. The BCS group was older (Cohen’s d = 1.01, *p* = 0.024) and taller (r = −0.47, *p* = 0.033) and exhibited significantly higher severity of self-reported signs and symptoms related to central sensitisation (Cohen’s d = 1.29, *p* = 0.005), neuropathic pain (Cohen’s d = 1.01, *p* < 0.001), BPI-severity (r = −0.99, *p* < 0.001), and BPI-interference (r = −0.99, *p* < 0.001) compared to the HC group. Additionally, the BCS group had significantly lower quality of life across several domains (physical function (Cohen’s d = 1.64, *p* < 0.001), role function (r = 0.63, *p* = 0.007), social function (r = 0.52, *p* = 0.024), bodily pain (Cohen’s d = −1.77, *p* < 0.001), and general health (r = 0.73, *p* = 0.001) than the HC group. No statistically significant differences were observed for the other variables. Detailed results, including test statistics and effect sizes, can be found in Table 2.

In terms of diet quality indices, there were no significant differences in HEI-2015 scores between the groups (Table 3). The BCS group (Mean ± SD = 0.4 ± 1.1) had a lower DII score, indicating lower pro-inflammatory potential, than the HC group (Mean ± SD = 1.4 ± 1.2) (Cohen’s d = −0.90, *p* = 0.041).

Table 3 displays the results of differences in the components of the HEI-2015, showing that the sodium score (Cohen’s d = 1.12, *p* = 0.012) is the only component that is significantly higher in the BCS group (Mean ± SD = 4.4 ± 2.0) compared to the HC group (Mean ± SD = 2.2 ± 1.9). Since sodium is a moderation component of the HEI-2015, a higher score indicates lower intake, meaning the BCS group consumes less sodium than the HC group.

The results of differences in the DII’s components and water intake are provided in Table 3. Compared to HC, BCS showed significantly higher intake of omega-3 (r = −0.47, *p* = 0.039), vitamin B6 (Cohen’s d = 0.88, *p*= 0.040), vitamin B12 (Cohen’s d = 1.10, *p* = 0.013), vitamin A (r = −0.58, *p* = 0.008), carotene (r = −0.52, *p* = 0.020), vitamin D (r = −0.52, *p* = 0.020), and magnesium (Cohen’s d = 0.45, *p* = 0.036) (Table 3). Both groups’ intake for the other macro- and micronutrients and water were similar.

The findings from the correlation analysis, detailing the relationship between dietary factors and pain outcomes in BCS experiencing chronic pain, are presented in Table 4. While overall indices did not reveal any correlations with pain outcome measures, a number of their components exhibited significant moderate to strong associations with pain outcomes.

Among the components of HEI-2015, no significant relations were found between any of the HEI-2015 dietary components and TS, OA, PPT-chest, CSI, and BPI severity. However, four of the thirteen components showed significant but moderate associations with other pain outcomes, with correlation coefficients ranging from −0.495 to 0.696 (Table 4). The strongest positive correlation was found between sodium and EPT (r = 0.696), while the strongest negative correlation was observed between total vegetables and CSI (r_s_ = −0.495).

Moreover, while there was no association found between any of the DII dietary components and PPT-chest, 15 out of 28 components showed significant moderate to strong correlations with other pain outcomes, with correlation coefficients ranging from −0.750 to 0.661 (Table 4). The strongest positive correlation was found between vitamin C and OA (r = 0.661), while the strongest negative correlation was observed between vitamin B3 and TS (r = −0.750).

No statistically significant relationship was observed between water consumption and pain outcomes.

## 4. Discussion

This is, to our knowledge, the first study investigating the differences in diet quality and dietary factors between BCS with chronic pain and HC. Our findings revealed that BCS suffering from chronic pain had a more anti-inflammatory diet (i.e., lower DII scores) compared to the HC group. Moreover, BCS demonstrated higher intakes of omega-3, vitamin B6, vitamin B12, vitamin A, carotene, and vitamin D, as well as magnesium, while having lower sodium intake compared to HC. In addition, correlation analysis between dietary outcomes and pain outcomes in BCS unveiled many moderate to strong associations, highlighting the complex relationships between dietary factors and chronic pain experiences within this specific population.

A more anti-inflammatory dietary pattern observed in the BCS with chronic pain corresponds to higher consumption of omega-3, vitamin B6, vitamin B12, vitamin A, carotene, vitamin D, and magnesium, all of which are assumed to have anti-inflammatory properties, while sodium consumption, which is generally known to provoke inflammatory responses in the body [69], was lower in the BCS. These findings align with recommendations from the World Cancer Research Fund (WCRF), the American Institute for Cancer Research (AICR), and the American Cancer Society (ACS), which advocate for maintaining a healthy body weight, staying physically active, and following a healthy diet (like rich in vegetables, fruits, and whole grains) for long-term disease-free living and improved survival after cancer [70,71]. Given the strong motivation among cancer survivors to improve their treatment outcomes, quality of life, and overall survival through healthy eating and lifestyle choices [72], it is not surprising that BCS in this study adopted a dietary pattern with anti-inflammatory potential.

Pain outcomes (except PPT-chest) exhibited several diverse correlations with components of dietary indices. For HEI-2015 components, our findings suggest a potential association between a diet characterised by high consumption of specific food groups rich in anti-inflammatory and/or antioxidant compounds [20] and improved pain outcomes, namely whole fruits (associated with DN4 and BPI-interference). Conversely, certain components that are known linked to inflammation showed associations with increased pain outcomes including dairy (associated with BPI- interference) and total protein foods (associated with PPT-tibialis). Interestingly, higher sodium scores (indicating lower sodium consumption) were correlated with increased pain outcomes (EDT, EPT, and PPT-tibialis). These results may suggest that a dietary pattern with anti-inflammatory features might contribute to improved pain modulation, reduced pain severity, decreased pain interference, and, potentially, lower neuropathic pain.

The associations observed among DII components suggest a connection between nutrient intake rich in anti-inflammatory, antioxidant compounds, and neurotransmitters or pain modulators synthesis [13]. For example, in this study, dietary nutrients like total calorie intake, protein, total polyunsaturated fatty acids, omega 3, and iron are found to contribute to decreased pain sensitivity (EPT, TS, and PPT-tibialis). However, there are some interesting negative correlations between cholesterol intake and pain sensitivity (EPT) and positive correlations between anti-inflammatory macro-micronutrients, including omega 3, vitamin C, B vitamins, vitamin A, vitamin D, carotene and magnesium and pain symptoms (pain severity, pain interference, neuropathic pain, and central sensitisation). It is noteworthy that these findings contrast with available studies, which have linked low consumption of macro and micronutrients, including omega-3 fatty acids, vitamins B1, B3, B6, B12, and D, magnesium, zinc, and carotene to chronic neuropathic or inflammatory pain [73]. These contradictions could be due to differences in study populations, measurement methods, nutrient interactions, study design, or unaccounted-for confounding factors. Therefore, these varying outcomes emphasise the complexity of the relationship between dietary factors and pain and call for further consideration and research on individualised nutritional approaches.

Past studies showed that adhering to an anti-inflammatory diet, which includes increased fruit and cereal consumption while decreasing salt and pork intake, may improve chronic pain symptoms [69]. Moreover, adherence to the ACS guidelines is associated with improved health-related quality of life, including bodily pain, among BCS (mainly stage II–III) [74]. Similarly, increased adherence to overall WCRF/AICR recommendations following breast cancer diagnosis was linked to improved health status/quality of life, and a reduction in severe symptoms, including pain [75]. However, the same study also reported that of the three components of the WCRF/AICR guideline (BMI, physical activity, and diet), BMI and physical activity recommendations were associated with reduced pain, while dietary recommendations were not. In contrast, after being diagnosed with breast cancer, women with excellent diet quality showed significantly higher overall quality of life scores, including less bodily pain, compared to women with poor diet quality [19]. Additionally, according to another study, a higher adherence to the Mediterranean diet—which includes a low or moderate intake of red meat, milk, and sweets and a high intake of plant-based foods like fruits, vegetables, whole grains, legumes, nuts, and olive oil—may have a positive effect on the quality of life, including pain, for women with breast cancer [76]. Research indicates that low-carbohydrate and Mediterranean diets may be effective in managing chronic pain through their effects on oxidative stress and inflammation, although the role of dietary antioxidants is less certain, and overall, studies on these diets in relation to chronic pain are limited [77]. Moreover, given the various potential mechanisms through which diet may impact or interact with pain—ranging from diet quality and systemic inflammation regulation to weight loss, maintenance of healthy body weight, immune system regulation, and fostering a healthy gut microbiome environment [78]), it is crucial that future researcher explores this topic with more detail and larger sample sizes.

Strengths of the present study include the fact that it is the first study to investigate differences in dietary factors between BCS and HC and to explore the relationship between dietary outcomes and pain outcomes in BCS. A large number of quantitative sensory test paradigms (including testing pain sensitivity, offset analgesia, and conditioned pain modulation) combined in one study is rather unique for the field of pain studies in BCS. However, it is important to acknowledge several limitations. First, given the explorative nature of the study, the sample size was relatively limited. Nonetheless, every effort was made to recruit a representative sample to ensure maximal external validity. Second, due to the case-control design of the study, causation cannot be established, and generalising findings should be approached with caution. Additionally, the lack of certain sociodemographic data like ethnicity and socioeconomic status, which influence dietary quality including among cancer survivors [79], may have introduced bias due to their unequal distribution among participants. Furthermore, we did not collect detailed data on menopausal status, preventing us from analysing potential differences between menopausal and post-menopausal individuals, which could have significant effects on the results. Although a 3-day food diary is a commonly used method for dietary assessment, the addition of some objective measures (e.g., inflammatory markers and nutrient biomarkers) could increase the comprehensiveness of the assessment and allow for a more complete evaluation of individuals’ nutritional status and overall well-being. Furthermore, while our study assesses the association between certain macro- and micronutrients and pain outcomes, we were unable to examine the variability in nutrient combinations and dosages, which may interact to influence pain differently than individual nutrients. This variability may contribute to differences in observed outcomes. Future research should investigate these interactions to clarify their impact on chronic pain. The absence of 17 food parameters (like garlic, onion, turmeric, thyme, and flavonoids), which are mostly anti-inflammatory, from the DII calculations due to limitations in available data should be considered a limitation of the study. Lastly, this study focused on dietary differences between BCS with chronic pain and healthy controls without comparing BCS with chronic pain to those without pain. Future research should address this gap by examining dietary variations within the BCS population to gain insights into how diet may specifically influence chronic pain in this group. Acknowledging these limitations takes attention to the need to interpret findings cautiously and suggests potential improvements in future research.

## 5. Conclusions

While the HEI-2015 did not reveal significant differences between the groups, the DII indicated that BCS with chronic pain had significantly lower scores than HC, suggesting lower pro-inflammatory potential in their diet. Moreover, BCS had a higher intake of certain nutrients known for their anti-inflammatory properties. Lastly, while the overall dietary indices (HEI-2015 and DII) did not correlate with pain outcomes, several individual components within these indices showed significant moderate to strong associations with various pain outcomes, highlighting the many complex links between dietary factors and chronic pain in BCS.

Our findings revealed that more research is needed to explore the relationship between diet and chronic pain in BCS. To fill these gaps, future research on the effects of a healthy diet regimen on chronic pain in BCS should use more comprehensive methodological approaches, larger cohorts, and randomised controlled trials.

## Figures and Tables

**Table 1:** Characteristics of BCS (n = 12).

		Absolute Number (%)	Mean (Standard Deviation)
**Cancer stage**	**Stage 0–I**	3 (25%)	
	**Stage II–III**	5 (41.7%)	
	**Unknown**	4 (33.3%)	
**Affected site from cancer**	**Right**	8 (66.7%)	
	**Left**	4 (33.3%)	
**History of cancer treatment**	**Surgery**	10 (83.3%)	
	**Chemotherapy**	7 (58.3%)	
	**Radiotherapy**	9 (75%)	
	**Endocrine therapy**	4 (33.3%)	
	**Immunotherapy**	1 (8.3%)	
**Years after treatment**			4.1 (2.2)
**Dominant pain site**	**Right**	6 (50%)	
	**Left**	5 (41.7%)	
	**Cannot decide**	1 (8.3%)	
**Dominant pain site on affected site**	**Yes**	10 (83.3%)	
	**No**	2 (16.3%)	
	**Cannot Decide**	0 (%)	

**Table 2:** Comparison of the characteristics of BCS and HC.

	BCS (n = 12) Median (IQR)/Mean ± SD	HC (n = 12) Median (IQR)/Mean ± SD	U-Statistic/t-Statistic	Effect Size (r/Cohen’s d)	*p*-Value
**Age (years)**	52.8 ± 6.3	47.5 ± 3.8	t = 2.46	Cohen’s d = 1.01	0.024 *
**Weight (kg)**	72.0 (22.0)	72.1 (13.6)	U = 74.00	r = 0.03	0.932
**Height (cm)**	166.3 (7.5)	160.0 (8.5)	U = 35.50	r = −0.47	0.033 *
**Body Mass Index (kg/m^2^)**	27.0 (8.8)	29.4 (5.9)	U = 89.00	r = 0.22	0.347
**Body fat mass %**	37.2 ± 7.9	36.1 ± 4.9	t = 0.42	Cohen’s d = 0.17	0.677
**Fat Free Mass (kg)**	46.9 (8.8)	46.2 (6.5)	U = 73.00	r = 0.01	1.000
**Body water %**	44.6 ± 5.6	45.5 ± 3.5	t = −0.50	Cohen’s d = −0.20	0.622
**SF-36-Physical Function (/100)**	50.4 ± 23.2	82.1 ± 14.4	t = −4.02	Cohen’s d = −1.64	<0.001 *
**SF-36-Role Function (/100)**	0.0 (50.0)	100.0 (25.0)	U = 118.00	r = 0.63	0.007 *
**SF-36-Social Function (/100)**	63.0 (25.0)	75.0 (25.0)	U = 110.50	r = 0.52	0.024 *
**SF-36-Emotional Health (/100)**	33.0 (67.0)	83.5 (67.0)	U = 105.00	r = 0.44	0.060
**SF-36-Bodily Pain (/100)**	41.9 ± 9.8	73.5 ± 23.2	t = −4.34	Cohen’s d = −1.77	<0.001 *
**SF-36-Mental Health (/100)**	59.3 ± 12.5	69.0 ± 20.3	t = −1.40	Cohen’s d = −0.57	0.175
**SF-36-Vitality (/100)**	39.6 ± 17.5	55.0 ± 20.2	t = −2.00	Cohen’s d = −0.82	0.058
**SF-36-General Health (/100)**	37.5 (15.0)	65.0 (10.0)	U = 128.00	r = 0.73	0.001 *
**IPAQ-Total (min/week)**	4517.0 (8187.0)	1981.5 (1695.0)	U = 51.00	r = −0.27	0.242
**Electrical Detection Threshold (mA)**	3.5 ± 0.8	3.3 ± 0.3	t = 0.44	Cohen’s d = 0.18	0.664
**Electrical Pain Threshold (mA)**	10.3 ± 6.3	7.8 ± 2.3	t = 1.27	Cohen’s d = 0.52	0.225
**Temporal Summation**	13.8 (25.0)	20.0 (42.5)	U = 90.00	r = 0.23	0.319
**Electrical Offset Analgesia**	11.3 (13.1)	8.8 (18.0)	U = 58.00	r = −0.04	0.898
**PPT-Chest (kg/cm^2^)**	3.1 (1.0)	4.4 (1.6)	U = 105.00	r = 0.43	0.060
**PPT-Tibialis anterior (kg/cm^2^)**	7.2 ± 4.3	8.7 ± 2.3	t = −1.02	Cohen’s d = −0.41	0.321
**Central Sensitization Inventory (/100)**	50.0 ± 13.1	32.8 ± 13.6	t = 3.15	Cohen’s d = 1.29	0.005 *
**Douleur Neuropathique 4 (/10)**	4.8 ± 2.5	0.0	t = 6.65	Cohen’s d = 1.29	<0.001 *
**BPI-Severity (/10)**	4.1 (2.7)	0.0	U = 0.00	r = −0.99	<0.001 *
**BPI-Interference (/10)**	5.0 (4.6)	0.0	U = 0.00	r = −0.99	<0.001 *

IQR = Interquartile Range, SD = Standard Deviation, SF-36 = Short Form—36 Health Survey, Role Function = Role Limitations due to Physical Health, PPT = Pressure Pain Threshold, BPI = Brief Pain Inventory. Mann–Whitney U test (U-statistic) used for non-normally distributed variables. Independent *t*-test (t-statistic) used for normally distributed variables. Effect size is Cohen’s d for *t*-tests (mean difference) and r for Mann–Whitney U-tests (based on ranks). * Significant difference (*p* < 0.05) between BCS and HC, as indicated in bold.

**Table 3:** Differences in overall diet quality indices and their components.

	BCS (n = 12) Median (IQR)/Mean ± SD	HC (n = 12) Median (IQR)/Mean ± SD	U-Statistic/t-Statistic	Effect size (r_/_Cohen’s d)	*p*-Value
**Differences in overall diet quality indices based on HEI-2015 index**					
Total HEI-2015 score (/100)	61.6 ± 11.3	61.6 ± 9.0	t = 0.013	Cohen’s d = 0.01	0.990
Total DII Score (−5.5–+5.5)	0.4 ± 1.1	1.4 ± 1.2	t = −2.18	Cohen’s d = −0.90	0.041 *
**Differences in Healthy Eating Index-2015 Components**					
**Adequacy Components**					
Total fruits (/5)	3.5 ± 0.6	3.8 ± 1.6	t = −0.59	Cohen’s d = 0.24	0.563
Whole fruits (/5)	4.9 (0.8)	5.0 (0.0)	U = 100.00	r = 0.40	0.114
Total vegetables (/5)	4.7 (0.6)	5.0 (0.5)	U = 104.00	r = 0.44	0.068
Greens and beans (/5)	1.8 (3.1)	3.4 (4.9)	U = 82.50	r = 0.14	0.551
Whole grains (/10)	4.6 (5.5)	9.4 (10.0)	U = 91.00	r = 0.25	0.291
Dairy (/10)	8.6 (2.4)	4.6 (5.1)	U = 40.00	r = −0.41	0.068
Total protein foods (/5)	4.0 (1.6)	5.0 (0.2)	U = 100.50	r = 0.41	0.101
Seafood and plant proteins (/5)	1.7 (1.9)	5.0 (4.6)	U = 101.00	r = 0.38	0.101
Fatty acid ratio (/10)	3.7 ± 2.8	3.6 ± 3.0	t = 0.08	Cohen’s d = 0.03	0.941
**Moderation Components**					
Refined grains (/10)	7.50 (5.29)	6.6 (3.9)	U = 54.50	r = −0.23	0.319
Sodium (/10)	4.43 ± 2.03	2.2 ± 1.9	t = 2.74	Cohen’s d = 1.12	0.012 *
Added sugar (/10)	9.66 (1.35)	10.0 (2.0)	U = 82.50	r = 0.14	0.551
Saturated fats (/10)	3.55 ± 2.00	4.9 ± 2.7	t = −1.34	Cohen’s d = 0.55	0.193
**Differences in Dietary Inflammatory Index (DII) Components**					
Kcal intake	1543.2 (606.3)	1545.2 (506.4)	U = 55.00	r = −0.22	0.347
Protein (g)	76.3 ± 18.1	64.6 ± 17.7	t = 1.60	Cohen’s d = 0.66	0.123
Total fat (g)	71.4 (20.3)	60.2 (40.0)	U = 50.00	r = −0.27	0.219
Total carbohydrate (g)	185.8 ± 46.3	193.6 ± 54.3	t = −0.38	Cohen’s d = 0.16	0.707
Alcohol (g)	0.0 (0.0)	0.0 (11.5)	U = 66.00	r = −0.22	0.755
Caffeine (g)	0.2 ± 0.1	0.1 ± 0.1	t = 1.08	Cohen’s d = 0.40	0.290
Fiber (g)	19.2 ± 4.4	20.4 ± 5.6	t = −0.54	Cohen’s d = −0.22	0.592
Cholesterol (mg)	312.9 ± 116.5	321.9 ± 171.3	t = −0.15	Cohen’s d = −0.06	0.881
Fatty acids, total saturated (g)	25.6 (8.5)	20.5 (15.5)	U = 57.00	r = −0.19	0.410
Fatty acids, total monounsaturated (g)	27.6 ± 7.1	26.6 ± 9.6	t = 0.29	Cohen’s d = 0.12	0.777
Fatty acids, total polyunsaturated (g)	12.2 (10.0)	11.9 (7.5)	U = 67.00	r = −0.07	0.799
Omega-3	4.3 (23.0)	1.2 (0.7)	U = 36.00	r = −0.47	0.039 *
Omega-6	12.3 ± 5.4	11.2 ± 3.5	t = 0.57	Cohen’s d = 0.23	0.577
Vitamin C (mg)	100.2 ± 45.3	94.5 ± 61.1	t = 0.26	Cohen’s d = 0.11	0.799
Vitamin B1—Thiamin (mg)	1.3 ± 0.2	1.2 ± 0.3	t = 0.14	Cohen’s d = 0.07	0.894
Vitamin B2—Riboflavin (mg)	2.0 (0.5)	1.6 (1.0)	U = 48.00	r = −0.31	0.178
Vitamin B3—Niacin (mg)	20.1 ± 5.4	17.7 ± 6.7	t = 0.97	Cohen’s d = 0.40	0.342
Vitamin B6 (mg)	2.2 ± 0.7	1.6 ± 0.6	t = 2.18	Cohen’s d = 0.88	0.040 *
Vitamin B9—Folic acid (mcg)	100.1 ± 54.4	100.4 ± 34.0	t = −0.02	Cohen’s d = −0.01	0.987
Vitamin B12—Cobalamin (mcg)	5.1 ± 2.6	2.8 ± 1.5	t = 2.70	Cohen’s d = 1.10	0.013 *
Vitamin A, RAE (mcg_RAE)	658.7 (74.0)	428.7 (207.0)	U = 27.00	r = −0.58	0.008 *
Carotene, beta (mcg)	3273.4 (1952.6)	2220.6 (2647.7)	U = 32.00	r = −0.52	0.020 *
Vitamin E, alpha-tocopherol (mg)	8.3 ± 3.1	6.9 ± 1.9	t = 1.32	Cohen’s d = 0.54	0.202
Vitamin D (D2 + D3) (mcg)	4.7 (2.2)	2.6 (2.7)	U = 32.00	r = −0.52	0.020 *
Iron (mg)	11.7 ± 1.9	11.5 ± 3.3	t = 0.22	Cohen’s d = 0.09	0.829
Magnesium (mg)	305.6 ± 45.5	262.1 ± 49.6	t = 2.24	Cohen’s d = 0.91	0.036 *
Zinc (mg)	9.9 ± 2.4	8.8 ± 2.4	t = 1.11	Cohen’s d = 0.45	0.279
Selenium (mcg)	94.1 ± 31.1	97.0 ± 28.8	t = −0.24	Cohen’s d = −0.10	0.816
**Additional component**					
Water (g)	2439.4 ± 634.9	2359.9 ± 692.4	t = 0.29	Cohen’s d = 0.12	0.772

IQR = Interquartile Range, SD = Standard Deviation. Mann-Whitney U test (U-statistic) used for non-normally distributed variables. Independent *t*-test (t-statistic) used for normally distributed variables. Effect size is Cohen’s d for *t*-tests (mean difference) and r for Mann-Whitney U-tests (based on ranks). * Significant difference (*p* < 0.05) between BCS and HC, as indicated in bold.

**Table 4:** Associations between dietary factors and pain outcome measures in BCS.

	EDT (n = 12)	EPT (n = 12)	TS (n = 12)	OA (n = 12)	PPT-Chest (n = 12)	PPT-Tibialis (n = 12)	CSI (n = 12)	DN4 (n = 12)	BPI-Severity (n = 12)	BPI-Interference (n = 12)
Total HEI-2015 score (/100)	0.021	0.040	0.006	0.413	−0.172	−0.058	−0.426	−0.329	0.282	−0.167
Total DII Score (−5.5–+5.5)	0.080	0.201	0.276	−0.588	0.281	0.386	0.379	0.267	−0.036	0.215
**Healthy Eating Index-2015 Components**										
Total fruits (/5)	0.075	0.216	0.045	0.186	0.063	0.257	−0.098	−0.326	−0.098	−0.426
Whole fruits (/5)	−0.083	−0.052	0.342	0.161	0.271	0.153	−0.249	−0.495 *	−0.359	−0.417 *
Total vegetables (/5)	−0.336	−0.397	−0.096	0.071	0.036	−0.485	−0.238	−0.222	0.097	0.138
Greens and beans (/5)	−0.448	−0.427	−0.194	0.287	−0.258	−0.452	−0.275	−0.204	0.110	0.047
Whole grains (/10)	−0.023	0.722	0.053	0.580	0.060	−0.167	−0.308	−0.194	0.169	−0.117
Dairy (/10)	−0.245	−0.075	0.222	0.046	−0.324	−0.302	0.170	0.383	0.364	0.435 *
Total protein foods (/5)	−0.098	−0.096	−0.292	0.065	0.179	0.428 *	−0.402	−0.384	−0.275	−0.372
Seafood and plant proteins (/5)	−0.177	−0.109	−0.164	0.204	−0.165	−0.277	−0.288	−0.248	−0.015	−0.235
Fatty acid ratio (/10)	0.035	0.098	0.090	−0.208	−0.087	−0.003	−0.367	−0.275	0.251	−0.033
Refined grains (/10)	−0.024	0.023	−0.037	0.012	−0.028	−0.149	−0.031	0.147	0.244	0.145
Sodium (/10)	0.591 *	0.696 *	0.320	−0.161	0.057	0.651 *	−0.231	0.018	0.353	−0.133
Added sugar (/10)	−0.136	−0.116	−0.075	−0.361	0.053	0.025	−0.197	−0.166	−0.069	−0.134
Saturated fats (/10)	−0.072	0.068	0.128	0.302	0.010	−0.116	−0.556	−0.312	0.140	−0.313
**Dietary Inflammatory Index Components**										
Kcal intake	−0.321	−0.437 *	−0.390	−0.042	−0.291	−0.517 **	0.396	0.189	0.126	0.258
Protein (g)	−0.186	−0.472	−0.669 *	0.562	−0.370	−0.464	0.382	0.009	−0.294	0.026
Total fat (g)	−0.166	−0.508	−0.469	0.098	−0.088	−0.419	0.324	−0.040	−0.040	0.360
Total carbohydrate (g)	−0.166	−0.367	−0.223	0.491	−0.180	−0.322	0.230	0.148	−0.193	0.074
Alcohol (g)	0.060	0.136	0.075	−0.259	0.166	0.136	−0.196	−0.194	−0.193	−0.193
Caffeine (g)	0.535	0.556	−0.082	−0.486	−0.041	0.603 *	0.205	0.420	−0.068	0.016
Fiber (g)	−0.160	−0.214	−0.218	0.568	−0.096	−0.357	−0.280	−0.177	−0.023	−0.227
Cholesterol (mg)	−0.292	−0.586 *	−0.407	0.103	−0.067	−0.416	0.323	−0.209	−0.171	0.258
Fatty acids, total saturated (g)	−0.162	−0.482	−0.429	0.159	−0.033	−0.327	0.550	0.169	−0.214	0.338
Fatty acids, total monounsaturated (g)	−0.042	−0.187	−0.047	−0.052	0.100	−0.160	−0.282	−0.302	0.239	0.182
Fatty acids, total polyunsaturated (g)	−0.134	−0.315	−0.500 *	0.129	−0.179	−0.172	0.197	0.068	0.064	0.152
Omega-3	−0.278	−0.388	−0.422 *	0.162	−0.370	−0.385	0.114	0.291	0.425 *	0.480 *
Omega-6	−0.141	−0.403	−0.534	0.014	−0.162	−0.395	0.441	0.060	−0.100	0.293
Vitamin C (mg)	−0.069	−0.196	−0.182	0.661 *	0.066	−0.137	−0.062	−0.006	0.021	−0.077
Vitamin B1—Thiamin (mg)	−0.164	−0.533	−0.484	0.340	−0.132	−0.386	0.432	0.099	−0.186	0.283
Vitamin B2—Riboflavin (mg)	0.107	−0.090	−0.451	−0.104	−0.127	−0.069	0.582 *	0.268	−0.322	0.116
Vitamin B3—Niacin (mg)	0.105	−0.237	−0.750 **	0.628 *	−0.375	−0.345	0.234	0.182	−0.235	−0.014
Vitamin B6 (mg)	0.402	0.179	−0.460	0.340	−0.294	−0.061	−0.098	0.214	−0.041	−0.116
Vitamin B9—Folic acid (mcg)	−0.053	−0.008	0.034	−0.361	−0.155	−0.173	0.254	0.014	−0.258	−0.029
Vitamin B12—Cobalamin (mcg)	0.424	0.354	−0.045	0.308	−0.339	0.114	−0.016	0.140	−0.073	−0.265
Vitamin A, RAE (mcg_RAE)	0.245	0.233	−0.176	0.227	−0.112	−0.047	0.214	0.479 *	0.564 **	0.545 **
Carotene, beta (mcg)	0.218	0.213	0.022	0.307	−0.195	−0.228	0.177	0.355	0.470 *	0.417 *
Vitamin E, alpha-tocopherol (mg)	0.227	0.409	0.156	−0.272	0.142	0.147	−0.330	−0.149	0.039	−0.300
Vitamin D (D2 + D3) (mcg)	−0.010	−0.119	−0.200	0.003	−0.264	−0.293	0.272	0.460 *	0.451 *	0.492 *
Iron (mg)	−0.291	−0.622 *	−0.568	0.255	−0.161	−0.532	0.368	0.048	−0.168	0.304
Magnesium (mg)	0.083	−0.169	−0.496	0.623 *	−0.098	−0.171	0.082	0.023	−0.038	−0.059
Zinc (mg)	0.492	0.313	−0.313	−0.190	0.084	0.449	0.394	0.400	−0.152	0.080
Selenium (mcg)	−0.207	−0.557	−0.680 *	0.117	−0.208	−0.506	0.425	−0.046	−0.257	0.119
**Additional component**										
Water (g)	0.159	0.110	−0.149	−0.173	0.010	0.329	0.283	0.188	−0.132	0.079

HEI-2015: Healthy Eating Index-2015, DII: Dietary Inflammatory Index, EDT = Electrical Detection Threshold, EPT = Electrical Pain Threshold, TS= Temporal Summation, OA = Electrical Offset Analgesia, PPT = Pressure Pain Threshold, CSI= Central Sensitization Inventory, DN4 = Douleur Neuropathique 4, BPI = Brief Pain Inventory. * Correlation is significant at the 0.05 level (2-tailed)/** Correlation is significant at the 0.01 level (2-tailed). Black: Pearson correlation for normally distributed variables; Blue: Spearman’s rho for non-normally distributed variables. Significant correlations were indicated in bold.

## Data Availability

The data presented in this study are available on request from the corresponding author due to confidentiality and privacy restrictions in accordance with ethical guidelines and data protection laws.
